# The relationship between sense of community and general well-being of Chinese older adults: A moderated mediation model

**DOI:** 10.3389/fpsyg.2022.1082399

**Published:** 2023-01-06

**Authors:** Tingting Huang, Houchao Lyu, Xueying Chen, Jia Ren

**Affiliations:** ^1^Southwest University, Chongqing, China; ^2^Nanning Normal University, Nanning, Guangxi, China; ^3^Mianyang Teachers’ College, Mianyang, Sichuan, China

**Keywords:** sense of community, community participation, social support, general well-being, older adults, China

## Abstract

As China becomes an aging society, the impacts of the aging population on the social meso domain, namely, the community level, have received increasing attention in recent years. However, relevant studies are limited. With the assumption that regular community participation positively influences well-being, this study investigated the mediating role of community participation between the sense of community and the general well-being of Chinese older adults and the moderating role of social support. A questionnaire survey was conducted with a valid sample size of 566 participants aged 60 and above in the urban communities of Chongqing, Chengdu, and Zunyi in southwest China. Moderated mediation models were constructed to explore factors related to the well-being of older adults, finding that encouraging community participation can improve the general well-being of older adults and build a better society in Chinese cities. The main findings of this study are as follows: (1) a sense of community significantly and positively relates to community participation and general well-being; (2) community participation partially mediates the relationship between sense of community and general well-being; and (3) each pathway through which sense of community influences older adults’ general well-being is moderated by social support.

## 1. Introduction

Currently, China is in a period of social transformation, and the problem of its aging population is becoming increasingly serious. According to the seventh national census in 2020, the number of people aged 60 and above was about 260 million, accounting for 18.7% of the Chinese population (National Bureau of Statistics of China, 2021). It is predicted that by 2050, the population aged 60 and above will reach 492 million (34.8%). More and more policymakers and professional researchers are concerned about how to deal with the aging problem and how to ensure active aging and happy in later life. According to Maslow’s Hierarchy of Needs, when lower-level needs such as security and physiological needs are met, people begin to seek higher-level needs such as belonging, respect, and self-actualization, and older adults are no exception (Cooper et al., 2017). For example, a sense of community is a resource enabling people to meet their physical and psychological needs. When the community meets the needs of individuals, community members are more willing to participate in community activities, improve the community environment, and remain stable residents in the community over time (McMillan and Chavis, 1986; Nowell and Boyd, 2010).

Sense of community (SOC), a core concept in community psychology, is defined as “the sense that a person is part of a ready-made, mutually supportive network of relationships” (Sarason, 1974). SOC can be conceptualized in two dimensions: geographical and relational factors and individual-level and community-level factors, both of which can influence the SOC (Sagy et al., 1996). Research on SOC involves the health of a community, as well as the level of community or social participation, social well-being, academic success, empowerment, social cohesion, belongingness, and individual needs being met within a community setting (Chipuer and Pretty, 1999; Cicognani et al., 2008; Jacobs and Archie, 2008; Talò et al., 2014). Scholars agree that a higher level of SOC can positively impact communities and individuals (Talò et al., 2014). However, only a few studies quantify the effects of community participation on SOC and well-being. To address this research gap, this paper tends to estimate the mediating effect of community participation (CP), between SOC and general well-being (GWB), and social support (SS), which moderate the mediating effect above.

Previous studies found that older adults have a higher level of SOC than young and middle-aged adults, and that SOC increases with age, especially with old age (Jorgensen et al., 2010). Older adults, especially those with limited mobility, spend most of their time in local communities. According to the social resource theory (Lin, 2001), SOC can be considered as an intangible resource that can meet people’s physical and psychological needs, thereby increasing the likelihood of increased participation in community activities (Mak et al., 2009). SOC based on the theoretical model of needs has been empirically supported at two individual levels: mental health and community participation. For example, a psychological SOC has been found to be a predictor of volunteerism in older adults (Omoto and Packard, 2016). Researchers (Liu et al., 2017) also found that SOC is enhanced by participation in community activities. Therefore, SOC, as an aspect of social relationships, is important for an individual’s GWB.

Social support refered to the material and moral support that individuals receive from social relationships such as those with relatives, friends, colleagues, or group organizations (Liu and Huang, 2010). The authors suggested that good social support can play a positive role when individuals experience emergencies and unexpected situations, helping individuals to move beyond a brief period of low mood. Xiao (1994) classified social support as subjective support, objective support, and support utilization. According to Lin and Yeh (2014), social support affects individuals’ cognitive evaluations, thereby improving their adaptability to the environment. Social support makes individuals believe they are cared for and cared about, thereby reducing their psychological needs (Chen and Zhang, 2021). The physical and mental health of older adults is related to social support. When social support and relationship networks shift, the physical and mental health of older adults might be affected. Especially under the influence of the Chinese mainstream social thinking that “old and frail, unable to take care of themselves and in need of social assistance,” elders gradually develop psychological dependence and behavioral inaction, and social support becomes the emotional guarantee and spiritual support to maintain the community participation of the elderly (Chen, 2014).

Emotional social support such as encouragement, support, understanding, and respect can provide emotional comfort and spiritual support for a greater sense of well-being in older adults. According to the social–emotional choice theory, individuals place more emphasis on companionship and emotional social support systems in later life, thereby diverting individuals’ attention and energy to their own physical and mental health needs (Chu et al., 2010). Meta-analysis has found that social support and subjective well-being are moderately correlated, confirming the positive effects of social support (Yao et al., 2018). Tovel and Carmel (2014) found that integrated social support can indirectly affect the subjective well-being of older adults *via* coping resources (i.e., a strong sense of community) and coping patterns (i.e., community participation). This finding is consistent with the findings of Liddle et al. (2014), which found that older adults, especially as they become increasingly frail, are able to receive support and feel happy in the environment they are accustomed to.

Community participation, as one of the main components of active aging, advocates that older adults should engage in society in a holistic manner, a view that is becoming a common consensus in addressing population aging globally. For example, Fowler and Christakis (2008) found that increased community participation behaviors and higher identity with community life affect residents’ evaluation of their Sense of community, thereby enhancing their well-being. The importance of community participation on older adults’ well-being was also documented by Au et al. (2020) in China. Jiang (2009) found that older adults are a major component of community participation, and involvement in community activities (i.e., volunteer activities and senior citizens clubs) can meet older adults’ social needs. By actively participating in community activities, older adults can increase opportunities to communicate with others, gain recognition, enhance psychological resilience, reduce feelings of helplessness, and gain a sense of self-worth and well-being.

Identifying feasible ways to encourage older adults to participate in community affairs and enhance their sense of well-being can help to identify the current situation of social support and community participation of older adults and enrich knowledge about their SOC and well-being. For example, Baker and Palmer (2006) found that longer participation and more activities are predictive of better well-being. A study in Taiwan found that participation in community activities enhances older adults’ sense of closeness and connectedness to other community members, helping them avoid feelings of rejection and isolation (Chen et al., 2015). Another study in Hong Kong found that participation in volunteer activities can improve the quality of life of older adults (Au, 2015). In mainland China, scholars found that older adults experienced a sense of accomplishment after being involved in various recreational activities, and participating in voluntary services (i.e., community policing patrols), enabling them to experience the realization of self-worth, meaningfulness, and fullness of life. These enhancements should therefore involve sustaining opportunities for developing and maintaining significant relationships, participating in the community in ways meaningful to them, and access to community and health services (Chaudhury et al., 2012; Wahl et al., 2012; Scharlach and Lehning, 2013).

**Figure 1:**
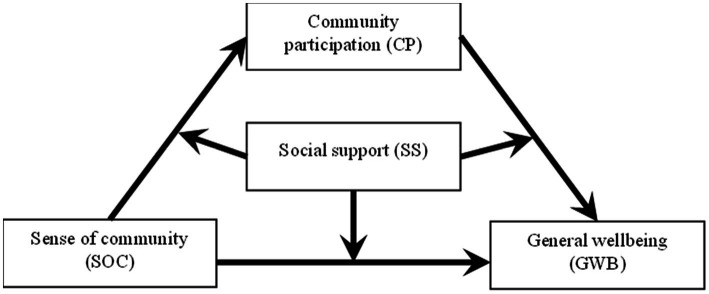
Conceptual framework.

Based on the above literature review, this study proposes a hypothetical model of the relationships among sense of community, social support, community participation, and general well-being of older adults (see Figure 1). This study conducted questionnaire surveys with older adults in southwest Chinese urban communities to test the model.

## 2. Methodology

### 2.1. Data collection

In 2022, 588 anonymous questionnaires were collected by online survey using convenience sampling. Posters in community centers were used to source respondents. The samples were collected from the urban communities of Chongqing, Chengdu, and Zunyi. All three cities are located in southwest China, an area considered a less developed region of the country. Participation was completely voluntary, and respondents could skip any questions they preferred not to answer. The survey consisted of five parts: the first section concerned participants’ sense of community; the second section measured older adults’ community participation; and the third and four sections collected respondents’ views on social support and general well-being, respectively. The last section collected the social and demographic information of the respondents, including age, gender, and monthly income. Questionnaires were sorted and checked to remove incomplete entries. After data cleaning, 566 valid survey entries remained, a return rate of 96.26%.

The gender and marital status composition of participants were identical to the census (National Bureau of Statistics of China, 2021). Out of 566 participants, 248 (43.82%) were male, and 318 (56.18%) were female. In terms of marital status, 450 were married, 11 were living together, 42 were unmarried, and 63 were divorced or widowed. In terms of socio-economic characteristics, however, participants in this study tended to be more educated and have a higher income than the national average. In terms of education attainment, 136 respondents had bachelor’s degrees and above, 312 were high school graduates, and 118 had completed junior high school and below. Monthly income was classified into four categories as follows: 40 participants earned less than 2,000RMB, 286 earned between 2,000 and 5,000RMB, 164 earned between 5,000 and 8,000RMB, and 76 earned between 8,000RMB, and above.

### 2.2. Data processing

After data cleaning, a series of descriptive analyzes, including analysis of variance, and Pearson’s correlation analysis, were performed using SPSS 25.0. Next, we used Hayes (2018) PROCESS macro (Model 4) to examine the mediating effect of the community participation on the relationship between the sense of community and the general well-being. In addition, we examined whether the social support moderated the mediation process with Hayes (2018) PROCESS macro (Model 59). Finally, the simple slope test was applied to explain the moderation effect. All variables were standardized before mediating and moderating effects were examined.

### 2.3. Measurements

#### 2.3.1. Sense of community index scale

The Sense of Community Index Scale, the second version of the Sense of Community Index scale (SCI) revised by McMillan (1996), is a quantitative measure of community sense, divided into four dimensions of membership, integration, satisfaction of needs, influence, and shared emotional connection, with a total of 24 items. Each item was scored on a 4-point scale, ranging from 1 (strongly disagree) to 4 (strongly agree). This questionnaire has been used in multiple Chinese studies (Ma et al., 2022), showing good reliability and validity. The internal consistency coefficient of the sense of community index scale in this study was 0.978.

#### 2.3.2. The community participation questionnaire

The six-item Community Participation Questionnaire for Urban Residents (Lin and Yeh, 2014) measured residents’ community participation in public affairs and private affairs. Each question of this scale was marked either “yes” (1 point) or “no” (2 point). For example, “In the past 3 months, have you participated in community public affairs maintenance activities?”. This scale had been applied in various empirical studies in China (Lin and Yeh, 2014; Greenfield et al., 2016). The internal consistency coefficient of community participation in this study is 0.98.

#### 2.3.3. Social support rating scale

This 10-item scale was designed by Xiao (1994) to measure social support in China. It includes three dimensions of social support, namely objective support, subjective support, and utilization of social support. From item-1 to item 5, each item was scored from 1 (no support) to 4 (full support) points respectively, while item 6 and item 7 were scored 0 points if respondent answered “no source” and several points if answered “the following sources”. This scale had been applied in various empirical studies in China (Ren et al., 2022). The internal consistency coefficient of the social support scale in this study was 0.761.

#### 2.3.4. General well-being schedule

This scale was proposed by Fazio (1977), and modified by Duan (1996) to evaluate subjects’ statements of well-being in China (Wu et al., 2010). This scale included six factors, namely worry about health, energy, satisfaction and interest in life, depression or pleasant mood, control of emotions and behavior, and relaxation and tension (anxiety) factors, with a total of 25 items. Item 24 was deleted because 352 respondents did not answer this question. Besides, after removing item 24, the overall reliability coefficient of the scale (0.937) was higher than the original (0.91). The internal consistency coefficient of the scale was 0.91 for men and 0.95 for women, with a retest reliability of 0.85 in this study.

## 3. Model results

### 3.1. Descriptive analysis and common method bias test

To avoid common method bias, reverse scoring of items was applied during the implementation of the study, while Harma’s (Fang and Sun, 2014) one-way method was adopted to operate the common method bias test. After EFA of the data of all variables, it was found that the total number of factors with eigenvalues greater than 1 without rotation was 9, and the first-factor variance explained 37.83%, lower than the critical criterion of 40%. Therefore, the data of this study did not have serious common method bias. Descriptive statistics of model variables were listed as follows (Table 1).

**Table 1:** Descriptive statistics of means, SD, and Pearson’s correlations (*n* = 566).

	SS	GWB	CP	SOC
Social support (SS)	1			
General well-being (GWB)	0.348**	1		
Community participation (CP)	0.626**	0.400**	1	
Sense of community (SOC)	0.341**	0.339**	0.710**	1
MEAN	27.700	4.178	1.186	3.110
SD	6.627	1.001	0.800	0.756

**p < 0.01.

### 3.2. Test of mediating effect

**Figure 2:**
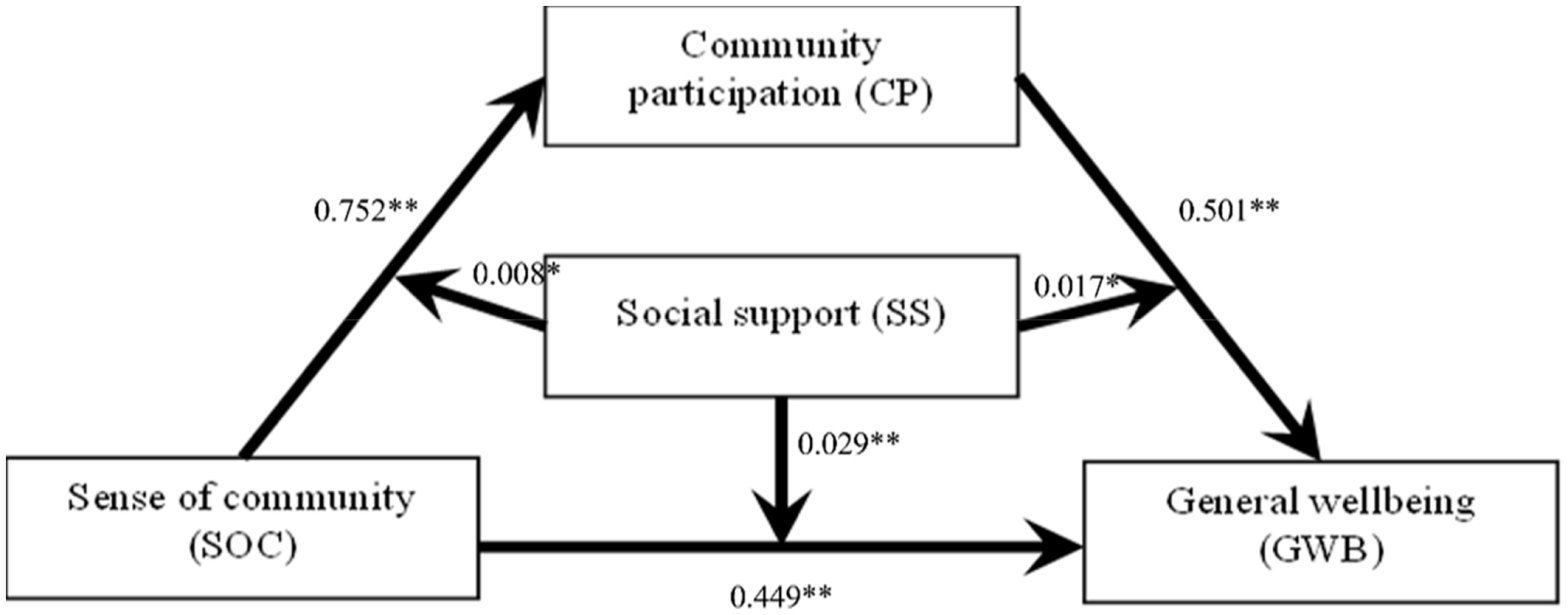
The moderated mediation model of social support and community participation. *p<0.05 and **p<0.01.

As shown in Figure 2, community participation partially mediates the relationship between a sense of community and general well-being. Each pathway through which sense of community influences older adults’ general well-being is moderated by social support.

The analysis of model results confirmed that CP mediates the relationship between SOC and GWB of older adults (see Tables 2, 3). The bootstrap 95% confidence interval of the mediating effect of community participation does not contain a 0 value. The direct effect was 0.146, [Bootstrap 95%, CI: (0.004, 0.288)], accounting for 32.52% of the total effect. The indirect effect was 0.303, accounting for 67.48% of the total effect [Bootstrap 95%, CI: (0.193, 0.422)], respectively. This indicates that community participation has a partial mediating effect between SOC and GWB.

**Table 2:** Model results (*N* = 566).

	GWB				CP				GWB			
	B	s.e.	*t*	*β*	*B*	s.e.	*t*	*β*	*B*	s.e.	*t*	*β*
Constant	2.781**	0.168	16.550	-	−1.152**	0.100	−11.467	-	3.246**	0.181	17.905	-
SOC	0.449**	0.053	8.551	0.339	0.752**	0.031	23.944	0.710	0.146*	0.072	2.016	0.110
CP									0.403**	0.068	5.892	0.322
*R* ^2^	0.115				0.504				0.166			
Adjusted *R*^2^	0.113				0.503				0.163			

*p<0.05 and **p<0.01.

**Table 3:** Results of mediating effect analysis (*N* = 566).

	Effect	BootSE	BootLLCI	BootULCI
Total effect	0.449	0.053	0.346	0.552
Direct effect	0.146	0.072	0.004	0.288
Indirect effect of CP	0.303	0.060	0.193	0.422

### 3.3. Test for moderated mediating effects

**Figure 3:**
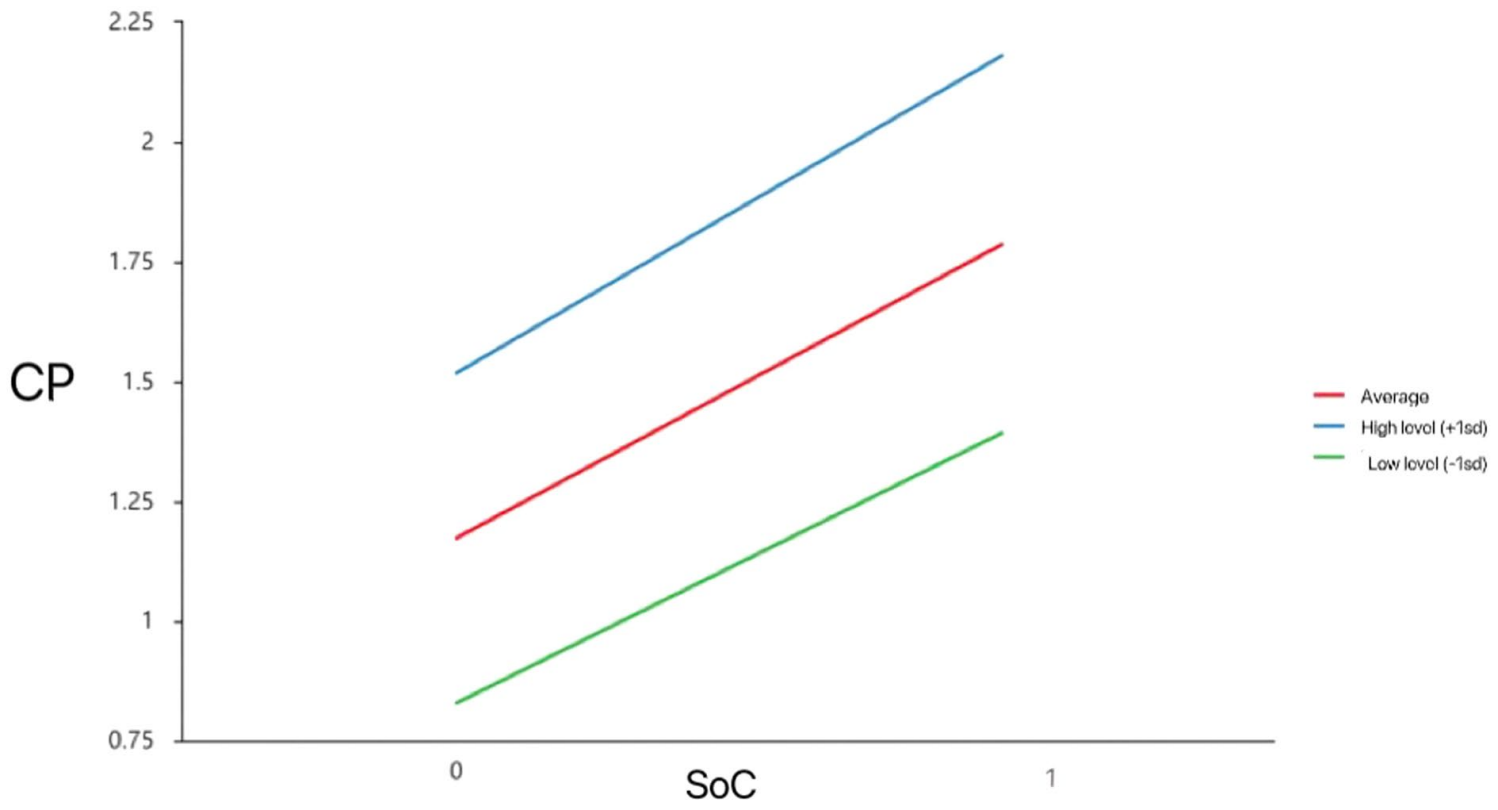
Model of test for simple slopes showing moderating influence of SS on association between SoC and CP.

First group of models measured the effects of SOC on older adults’ CP without the moderating effects of SS (Table 4). Dependent variable was CP, and independent variables were SOC and SS. According to the results in model 1-a, SOC was significantly related to CP (*t* = 23.944, *p* = 0.000), indicating that SOC has a significant influence on the CP of the elderly. As shown in model 1-c, the interaction term between SOC and SS was statistically significant (*t* = 2.032, *p* = 0.043), suggesting that the magnitude of the effect of the moderating variable (SS) on CP of the elderly is significantly different at different levels, as shown in the “Simple slope plot analysis (Figure 3).”

**Table 4:** Sense of community and community participation: moderating roles of social support.

	Model 1-a				Model1-b				Model 1-c			
	*B*	s.e.	*t*	*β*	*B*	s.e.	*t*	*β*	*B*	s.e.	*t*	*β*
Constant	1.186**	0.024	50.040	-	1.186**	0.019	61.410	-	1.173**	0.020	57.945	-
SOC	0.752**	0.031	23.944	0.710	0.595**	0.027	21.858	0.562	0.613**	0.029	21.404	0.579
SS					0.056**	0.003	16.924	0.435	0.055**	0.003	16.739	0.431
SOC*SS									0.008*	0.004	2.032	0.052
*R* ^2^	0.504				0.671				0.674			
Adjusted *R*^2^	0.503				0.670				0.672			
*F*	*F* (1,564) = 573.312**				*F* (2,563) = 574.949**				*F* (3,562) = 386.807**			
△*R*^2^	0.504				0.167				0.002			
△*F*	*F* (1,564) = 573.312**				*F* (1,563) = 286.437**				*F* (1,562) = 4.130*			

*p < 0.05, **p < 0.01.

**Figure 4:**
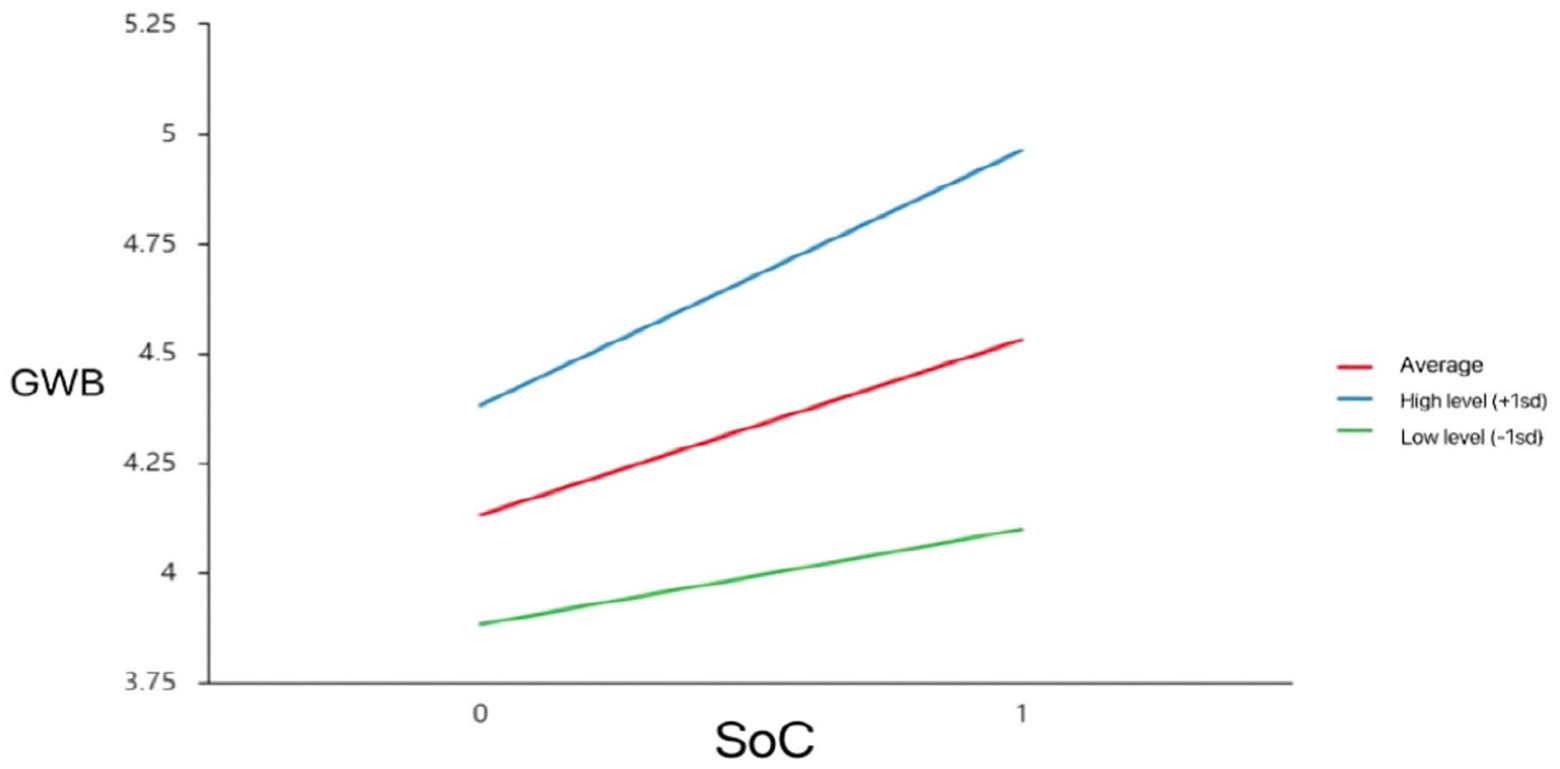
Model of test for simple slopes showing moderating influence of SS on association between SoC and GWB.

Second group of models (Table 5) measured the effects of SOC on GWB without considering the moderated effects of SS. As model 2-a shows, SOC was statistically significant (*t* = 8.551, *p* = 0.000), indicating that SOC has a significant influence on older adults’ GWB. According to model 2-c, the interaction term between SOC and SS was significant (*t* = 3.812, *p* = 0.000), meaning that the magnitude of the effect of the moderating variable (SS) on GWB is significantly different at different levels, as shown in the “Simple slope plot analysis (Figure 4).”

**Table 5:** Sense of community and general well-being: moderating roles of social support.

	Model 2-a				Model 2-b				Model 2-c			
	*B*	s.e.	*t*	*β*	*B*	s.e.	*t*	*β*	*B*	s.e.	*t*	*β*
Constant	4.178**	0.040	105.409	-	4.178**	0.038	109.141	-	4.131**	0.040	103.872	-
SOC	0.449**	0.053	8.551	0.339	0.330**	0.054	6.125	0.249	0.400**	0.056	7.096	0.301
SS					0.042**	0.007	6.453	0.263	0.040**	0.006	6.184	0.250
SOC*SS									0.029**	0.008	3.812	0.152
*R* ^2^	0.115				0.176				0.197			
Adjusted *R*^2^	0.113				0.173				0.192			
*F*	*F* (1,564) = 73.121**				*F* (2,563) = 60.016**				*F* (3,562) = 45.817**			
△*R*^2^	0.115				0.061				0.021			
△*F*	*F* (1,564) = 73.121**				*F* (1,563) = 41.642**				*F* (1,562) = 14.533**			

*p < 0.05, **p < 0.01.

**Figure 5:**
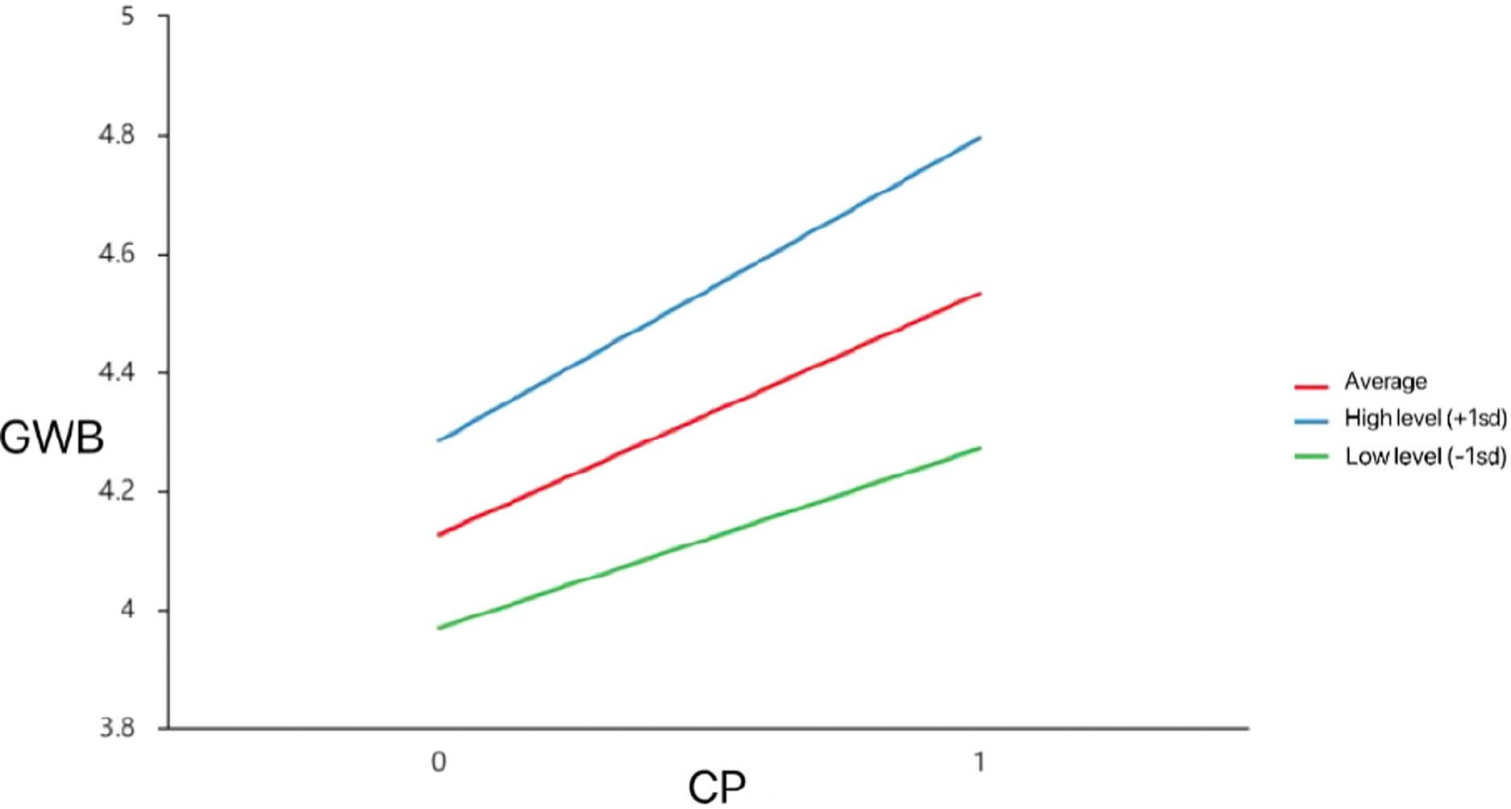
Model of test for simple slopes showing moderating influence of SS on association between CP and GWB.

The last set of models (models 3-a, 3-b, and 3-c) measured the mediated effects of SS between CP and GWB. As Table 6 shows, the independent variable (CP) was significant (*t* = 10.371, *p* = 0.000), indicating that CP refers to a significant influence relationship on GWB. The interaction term between CP and SS was statistically significant (*t* = 1.989, *p* = 0.047). This means that the magnitude of the effect of the moderating variable (SS) on the GWB of the elderly is significantly different at different levels, as shown in the “Simple slope plot analysis (Figure 5).”

**Table 6:** Community participation and general well-being: moderating roles of social support.

	Model 3-a				Model 3-b				Model 3-c			
	*B*	s.e.	*t*	*β*	*B*	s.e.	*t*	*β*	*B*	s.e.	*t*	*β*
Constant	4.178**	0.039	108.220	-	4.178**	0.038	109.133	-	4.126**	0.046	89.226	-
CP	0.501**	0.048	10.371	0.400	0.376**	0.061	6.117	0.3	0.407**	0.063	6.435	0.325
SS					0.025**	0.008	3.249	0.159	0.025**	0.008	3.222	0.158
CP*SS									0.017*	0.008	1.989	0.080
*R* ^2^	0.160				0.176				0.181			
Adjusted *R*^2^	0.159				0.173				0.177			
*F*	*F* (1,564) = 107.553**				*F* (2,563) = 59.964**				*F* (3,562) = 41.505**			
△*R*^2^	0.160				0.015				0.006			
△*F*	*F* (1,564) = 107.553**				*F* (1,563) = 10.553**				*F* (1,562) = 3.957*			

*p < 0.05, **p < 0.01.

According to Figures 3, 4, SoC had a significant positive effect on CP (i.e., one SD below the mean; *β*_simple_ = 0.564, *p* < 0.05) and GWB (i.e., one SD below the mean; *β*_simple_ = 0.217, *p* < 0.05). When the level of social support was high (i.e., one SD above the mean), the effect of SoC on CP (*β*_simple_ = 0.663, *p* < 0.05) and GWB increased (*β*_simple_ = 0.582, *p* < 0.05). That is, when older adults feel higher levels of social support, the SoC better predicts their GWB.

As shown in Figure 5, CP had a significant positive effect on GWB whatever when social support was low (i.e., one SD below the mean; *β*_simple_ = 0.303, *p* < 0.05), or the level of social support was high (i.e., one SD above the mean), the positive predictive effect of CP on GWB increased (*β*_simple_ = 0.511, *p* < 0.05).

## 4. Discussion

### 4.1. Mediating role of community participation

According to the model results, older adults’ sense of community was significantly and positively related to community participation and general well-being (Table 1). This finding suggests that older adults’ sense of community in urban China can strengthen their sense of responsibility and obligation for community affairs, as well as their perception of the common good of the community, which, in turn, affects their enthusiasm for community participation. In addition, community participation is also a process of continued socialization; that is, individuals’ transition from middle age to old age is a necessary stage and can be considered as the completion stage of individual socialization. This is consistent with Talò et al. (2014), who found that older adults face a transition in social and family roles after retirement, and those with a stronger sense of community can actively integrate into the social participation process, resulting in better psychological health and fewer negative emotions such as depression and anxiety (Millner et al., 2019).

This study also found that community participation had positive effects on the well-being of Chinese urban older adults, a finding in line with existing studies. For example, Hannah and Spencer (2012) confirmed that community participation, as an important component of an individual’s social functioning, can provide role support to maintain self-concept and bring happiness. Positive activity theory also emphasizes that older adults should actively participate in their community and continue past activities to gain positive emotions, enjoy life, and experience happiness (Bess et al., 2002). It argues that even after retirement, maintaining activity patterns is an important foundation enabling older adults to achieve a full and happy life and improve the quality and quality of life. It is a key path to active aging. In general, older adults have become an active and important force in the practice of community participation. Older adults can not only draw spiritual nourishment and cultivate their temperament through community participation, but also adjust and adapt to their changing roles physically and psychologically, improve their life satisfaction, increase their positive emotional experience, and reduce their negative emotional experience, thereby helping them to achieve a state of happiness in their later life (Lemon et al., 1972; Lampinen et al., 2006).

### 4.2. Moderating role of social support

The findings suggest that social support affects older adults’ general well-being through community participation as moderating variable, and that the three pathways of SoC-GWB, SoC-CP, and CP-GWB are enhanced at different moderating levels when older adults receive higher social support. As shown in Figure 5, regardless of the level of social support, community participation had a significant positive effect on GWB, and the effect of community participation on GWB increased if the level of social support increased. This is consistent with the existing empirical findings (Chen, 2014) that when older adults have adequate levels of social support, they are more willing to participate in community activities. For example, Chen (2014) argues that most older adults attribute their increased participation in social activities to social support rather than self-factors. At the same time, the social nature of human beings determines that their behavior will be influenced by the interpersonal relationships around them, and their opinions, attitudes, and behaviors will undoubtedly affect the level of social participation of older adults. As Long and Perkins (2007) say, People’s community participation behaviors are both a result of the growing abundance of community resources and they are able to meet people’s needs. Therefore, our future research will explore what kinds of community activities can enhance older adults’ social support, or how to facilitate their community participation behaviors through pathways that increase social support.

Nelson et al. (2016) found that active social roles, such as participation in community service and volunteer activities, are ways to achieve self-worth and enhance satisfaction and well-being. With adequate social support, older adults can have a better sense of community and are more likely to be motivated to participate in community affairs, and promoting a higher level of general well-being. This also confirmed in our findings that when older adults feel higher levels of social support, the sense of community better predicts their community participation and general well-being (Figures 3, 4). At the same time, community participation forms a positive interpersonal interaction and social support, factors which can relieve the psychological stress of older adults and enhance their confidence and ability to cope with life events. Therefore, older adults can experience higher levels of well-being by enhancing the social support they receive.

### 4.3. Limitations and future studies

There are some limitations that this study plans to address in future studies. First, due to funding and capacity constraints, a convenience sampling approach was adopted for data collection, resulting in inadequate data representation. Second, the educational attainment and income level of participants are higher than the national average, indicating that the empirical validity and ecological validity of the study results are limited. Finally, self-election bias might be a problem because the online survey was distributed through community centers, and only those who are willing to participate in community activities could access the questionnaire and were more likely to answer it.

In future studies, the sample should be expanded as much as possible, for example, by including a sample of rural older adults and a sample from more regions, to obtain more representative overall research data and obtain more generalizable research results. Second, a sophisticated research method should be adopted, either through before-and-after control or by conducting a longitudinal tracking study of research results and countermeasures to improve the validation of the analysis generalizability, whereby the applicability of the analysis and countermeasures can be improved. Finally, the study could continue to explore the factors affecting the happiness of the elderly from different dimensions and aspects.

## 5. Conclusion

According to our questionnaire survey, older adults in urban China are experiencing relatively high levels of general well-being. Our descriptive analysis reveals that female participants have a significantly higher level of community participation and sense of community than male participants. Moreover, older adults who are better educated and those with higher income levels tend to have better general well-being. A higher sense of community is an important factor in promoting community participation and enhancing the general well-being of older adults. Cultivating a sense of community among older adults is consistent with their motivation to “play their part and make a difference in their old age,” and is conducive to giving full play to their values in community participation. The social support of older adults contributes positively to their community participation and general well-being, and higher social support can promote and enhance their community participation, an important component of older adults’ physical and mental health and well-being with a significant potential impact on active aging.

## Data availability statement

The raw data supporting the conclusions of this article will be made available by the authors, without undue reservation.

## Author contributions

All authors listed have made a substantial, direct, and intellectual contribution to the work and approved it for publication.

## Funding

This work was supported by the Ministry of Education of Humanities and Social Science Project in China (No. 20YJA630011), the Digital Industrial College of Natural Resources–Development Fund Scheme (Guangxi, China), 2021 Chongqing Social Science Planning Key Project in China (No. 2021NDZD09), and the key project of Chongqing Education Science “14th Five-Year Plan” in 2021 of China (No. 2021-GX-003).

## Conflict of interest

The authors declare that the research was conducted in the absence of any commercial or financial relationships that could be construed as a potential conflict of interest.

## Publisher’s note

All claims expressed in this article are solely those of the authors and do not necessarily represent those of their affiliated organizations, or those of the publisher, the editors and the reviewers. Any product that may be evaluated in this article, or claim that may be made by its manufacturer, is not guaranteed or endorsed by the publisher.
