# Supplementary material for: The relationship between sense of community and general well-being of Chinese older adults: A moderated mediation model
Source: Front Psychol. 2023 Jan 6;13:1082399. doi: 10.3389/fpsyg.2022.1082399 (PMC9859671; doi:10.3389/fpsyg.2022.1082399)
Supplement: Supplementary file 1 [file Data_Sheet_1.docx]

**Urban residents community participation questionnaire**

1. In the past three months, have you participated in community public affairs maintenance activities?
2. in the past three months, have you participated in community public welfare activities?
3. in the past three months, have you participated in any community creation activities ?
4. in the past three months, have you participated in any community affairs supervision activities ?
5. in the past three months, have you participated in any community recreational activities ?
6. Did you participate in the last general election of the community committee?
