# Supplementary material for: The relationship between sense of community and general well-being of Chinese older adults: A moderated mediation model
Source: Front Psychol. 2023 Jan 6;13:1082399. doi: 10.3389/fpsyg.2022.1082399 (PMC9859671; doi:10.3389/fpsyg.2022.1082399)
Supplement: Supplementary file 2 [file Data_Sheet_2.docx]

**Social Support Rating Scale**

Instructions: The following questions are used to reflect the support you have received in the society. Please write them according to the specific requirements of each question and your actual situation. Thank you for your cooperation.

1. How many close friends do you have for support and help? (Choose only one item)

（1）Not a single one

（2）1-2

（3）3－5

(4）Six or more

2. Last Year You: (Choose only one item)

Living in a room away from family

Changes one‘s place of residence frequently and spends most of time with strangers

Stay with classmates, colleagues or friends

(4）I live with my family

3. You and your neighbors: (Choose only one)

（1）Never care about each other, just a nodding acquaintance

（2）Encounter difficulties may be a little concerned

（3）Some of the neighbors care about you

（4）Most of the neighbors care about you

1. You and colleagues: (Choose only one)

（1）Never care about each other, just a nodding acquaintance

(2）Encounter difficulties may be a little concerned

(3) Some of the neighbors care about you

(4) Most of the neighbors care about you

1. Support and care received from family members (tick the appropriate box

|  | None | Rare | Sometimes | Full support |
| --- | --- | --- | --- | --- |
| A.Husband and wife (lovers) |  |  |  |  |
| B.Parent |  |  |  |  |
| C.Sons and daught |  |  |  |  |
| D.Brothers and sisters |  |  |  |  |
| E.Other members (e.g., sister-in-law) |  |  |  |  |

1. In the past, financial support and practical help have come from:

Without any source

The following sources (optional)

A. Spouse; B. Other family members; C. Relatives; D. Colleagues; E. Workplace; F. Official or semi-official organizations such as parties and unions; G. Unofficial organizations such as religious and social groups; H. Others (please list)

7. In the past, you have been comforted and cared for in times of distress by:

（1）Without any source

(2) The following sources (optional)

A. Spouse; B. Other family members; C. Relatives; D. Colleagues; E. Workplace; F. Official or semi-official organizations such as parties and unions; G. Unofficial organizations such as religious and social groups; H. Others (please list)

8. How you talk about your troubles: (Choose only one)

（1) Never litigate to anyone

(2) Only confide in 1-2 people who are extremely close to you

(3) If a friend takes the initiative to ask you will say it

(4) Take the initiative to talk about your troubles to get support and understanding

1. The way you seek help when you are in trouble: (choose only one)

(1) Only rely on yourself and don't accept help from others

(2) Rarely ask for help from others

(3) Sometimes ask for help from others

(4) Always ask for help from family, relatives, friends and organizations when you are in trouble

1. For group (such as party organizations, religious organizations, trade unions, student unions, etc.) organizational activities, you: (choose only one)

(1) Never participate

(2) Occasionally participate

(3) often participate

(4) Participate actively and are active
